# Establishment and Application of Novel Hypoxia-driven Dual-reporter Model to Investigate Hypoxic Impact on Radiation Sensitivity in Human Nasopharyngeal Carcinoma Xenografts

**DOI:** 10.7150/jca.96378

**Published:** 2024-06-11

**Authors:** Jun Dong, Chengtao Wang, Tian Zhang, Xiaobi Yu, Haihua Peng, Zhenhua Xiao, Zhenyu Wang, Bixiu Wen

**Affiliations:** 1 Department of Radiation Oncology, The First Affiliated Hospital, Sun Yat-sen University, Guangzhou, Guangdong Province, 510080, China.; 2 Department of Clinical Oncology, The University of Hong Kong-Shenzhen Hospital, Shenzhen, Guangdong Province, 518048, China.; 3 Department of Radiation Oncology, The Fifth Affiliated Hospital, Sun Yat-sen University. Zhuhai, Guangdong, 519000, China.; 4 Department of Radiation Oncology, Affiliated Cancer Hospital & Institute of Guangzhou Medical University, Guangzhou, Guangdong Province, 510075, China.; 5 Department of Medical Physics and Radiation Oncology, Memorial Sloan-Kettering Cancer Center, NY, New York City, 10021, USA.

**Keywords:** Tumor hypoxia, DNA double strand breakage, Hypoxia-responsive element, HSV1-TKGFP, Non-homologous end joining, Synergistic anti-tumor effect

## Abstract

**Background:** Tumor hypoxia has been frequently detected in nasopharyngeal carcinoma (NPC) and is intently associated with therapeutic resistance. The aim of the study is to establish a clonogenically stable hypoxia-inducible dual reporter model and apply it to investigate the effect of tumor hypoxia on DNA double strand break (DSB) and synergistic effect of irradiation in combination with chemotherapy or targeted therapy.

**Methods:** The plasmid vector consisting of hypoxia response elements to regulate HSV1-TK and GFP genes, was constructed and stably transfected into human NPC cells. The expected clone was identified and validated by *in vivo* and *in vitro* assay. DSB repair was measured by γH2AX foci formation. Tumor growth delay assay and spatial biodistribution of various biomarkers was designed to investigate the anti-tumor effect.

**Results:** The system has the propensity of high expression of reporter genes under hypoxia and low to no expression under normoxia. Intratumoral biodistributions of GFP and classic hypoxic biomarkers were identical in poor-perfused region. Upon equilibration with 10% O_2_, the xenografts showed higher expression of hypoxic biomarkers. Cisplatin radiosensitized SUNE-1/HRE cells under hypoxia by suppressing DSB repair while the addition of PI3K/mTOR inhibitor further enhanced the anti-tumoral therapeutic efficacy. Combination of IR, DDP and NVP-BEZ235 exhibited most effective anti-tumor response *in vivo*. These observations underline the importance of dual reporter model for imaging tumor hypoxia in therapeutic study.

**Conclusions:** Our preclinical model enables the investigation of heterogeneous tumor hypoxic regions in xenograft tissues and explores the treatment efficacy of combinations of various therapeutic approaches to overcome hypoxia.

## Background

Hypoxia has been commonly observed in a variety of solid malignant neoplasms due to the rapid and uncontrolled cell proliferation and insufficient blood supply 1, 2. Oxygen can diffuse approximately 70µm from capillary where cells in the vicinity would be supposed to be normoxic. At greater distance, anoxic tumor cells become necrotic; while the hypoxic cells form a layer in between normoxic and anoxic cells 3, 4. Solid tumors with sub-regions of hypoxia are generally refractory to chemotherapy, radiotherapy (RT) and immunotherapy, which contributes to local tumor relapse and distant metastases in prostate cancer, non-small-cell lung cancer (NSCLC) and head and neck cancer 5-11.

The consequences of hypoxic tumor microenvironment (TME) develop via both hypoxia inducible factor (HIF)-canonical and non-canonical processes. The stability of HIF proteins under hypoxia activates the transcription of downstream genes, thus moderating the microenvironmental impetus in cancer resulting in an acidic, nutrient deprived and immune unfavorable TME 12.

Mechanism-driven therapy is an effective approach to improve therapeutic efficacy. Thus, detecting tumor hypoxia *in vivo* by a non-invasive method is important to uncover the mechanism of hypoxia-mediated radioresistance. Carbonic anhydrase 9 (CA9), an endogenous hypoxic marker, upregulated by HIF1α is predictive of hypoxia and the expression of CA9 is sustained at 72 h after reoxygenation 13. Pimonidazole is an exogenous hypoxic marker, exhibiting similar spatial distribution to CA9 in untreated tumors. Pimonidazole is cleared rapidly under normoxia and retained in hypoxia 14, 15. The establishment of hypoxia research models in prostate cancer and colorectal cancer containing a HSV1-TKGFP fusion gene which was transactivated by hypoxia-responsive promoter from EPO or VEGF gene has been reported to be sufficiently sensitive to determine hypoxia distribution *in vivo* by noninvasive microPET or invasive autoradiographic and fluoroscopic studies 16, 17.

Double DNA strand breakage (DSB) is the most severe damage caused by irradiation, which are primarily repaired via non-homologous end joining (NHEJ) and homologous recombination (HR) pathways. The highly reactive free radicals induced by ionizing radiation (IR) are unstable and required to react rapidly with oxygen to make lethal DNA breaks permanent. Conversely, DNA damage is repaired in absence of oxygen 18, 19. Both hypoxia and enhanced DNA damage repair are associated with radioresistant phenotype 2, 10, 20.

Nasopharyngeal carcinoma (NPC) is endemic in east and southeast Asia 21. Concurrent chemoradiotherapy (chemo-RT) is the standard of care for patients with locoregionally advanced NPC, while cisplatin (cis-diamminedichloroplatinum, DDP) is the preferred regimen of chemotherapy 22. Hypoxia has been commonly observed in primary and/or metastatic NPC during diagnostic imaging studies; while the tumors with hypoxic profile are associated with poor prognosis 23. ^18^F-FMISO (a tumor hypoxia tracer) PET/CT has been used for dose escalation in NPC 24. Radiosensitivity is strikingly enhanced in NPC cells by hampering NHEJ repair pathway 25, 26.

The study aims to establish and characterize a research model in NPC containing a hypoxic gene reporter imaging system that can accurately and reliably map hypoxia distribution within tumor tissue; and to investigate the effect of tumor hypoxia on DSB and synergistic effect of irradiation in combination with chemotherapy or PI3K/mTOR dual inhibitor in overcoming hypoxia-related radioresistance.

## Materials and methods

### Cells and reagents

SUNE1 cells, derived from a patient with undifferentiated NPC, were routinely cultured in RPMI-1640 supplemented with 10% fetal bovine serum (FBS), penicillin, and streptomycin (complete RPMI, cRPMI) at 37°C in 5% CO_2_. NVP-BEZ235 (Selleck Chemicals) was reconstituted in dimethyl sulfoxide (Sigma-Aldrich) and stored at -20°C for *in vitro* assay. DDP was bought from Sigma-Aldrich and reconstituted with Saline (1 mg/ml). It was kept at 4°C and used within 72 h after reconstitution.

### Generation of SUNE1-9xHRE-TKGFP (SUNE1/HRE) cells

The plasmid vector with hypoxia-driven gene expression fusion gene (9xHRE-HSV1-TKGFP) was described as previously 17. In brief, the plasmid vector consisted of hypoxia response elements (9xHRE) of 9 tandem repeats to regulate the fusion gene of human herpes simplex virus 1-thymidine kinase and enhanced green fluorescent protein (HSV1-TKGFP). The SUNE1/Parental (SUNE1/P) cells were stably transfected with 9xHRE-HSV1-TKGFP by calcium phosphate precipitation method. As the plasmid containing a constitutively expressed neomycin-resistance gene, the cells were maintained in cRPMI with 400 ug/mL G418 for 3 to 4 weeks after transfection.

The Neomycin-resistant cells were pooled and exposed to hypoxic condition (0.1% O_2_, 37°C) for 24 h and then sorted by fluorescence-activated cell sorting (FACS, Moflo cell sorter, Dako) for three times based on the hypoxia-induced GFP expression. To obtain clonogenically stable hypoxia-driven dual reporter gene expression research model with fusion gene of 9xHRE-HSV1-TKGFP, the third sorting cells were cloned into 96-well plates (3 cells/ml), incubated in 37°C, 5% CO_2_, 21% O_2_ condition. The individualized clone cells were collected and cultivated to further identify the ones with maximum hypoxia inducibility in terms of hypoxia-induced GFP expression by FACS and HSV1-TK gene expression using β ray scintillation detector (liquid scintillation counting; Perkin Elmer Life Sciences, Downers Grove, IL, USA) under hypoxic condition in an INVIVO2-400 Hypoxic Workstation (Biotrace, Inc., Cincinnati, OH, USA).

### Radiotracer assay for TKGFP expression *in vitro*

The level of hypoxia-induced TKGFP expression in SUNE1/P and SUNE1/HRE cells was assessed by using the ^14^C-2'-Deoxy-2'-fluoro-h-D-arabinofuranosyl-5-iodouracil (^14^C-FIAU) radiotracer assay as described 16, 17. Briefly, 2x10^5^ cells were transplanted at 6-well plates and were treated with 21% O_2_ or 0.1% O_2_ for different time intervals. The culture medium was replaced with medium containing ^14^C-FIAU (0.025 μCi/ml, Moravek Biochemicals, USA) and cultivated for 1 h. The cells were collected and rinsed with chilled PBS for three times. The cells were lysed with 1 mL of 0.3N NaOH/1% SDS and neutralized with 0.1 mL 3N HCl. The protein level was quantified by BSA assay. The uptake of ^14^C-FIAU was measured by Wallac 1410 liquid scintillation counter. The ^14^C-FIAU normalized to counting of radiosensitivity/mg protein and further normalized to fold according to the baseline.

### Mouse xenograft studies

Animal protocol was approved by Laboratory and Clinical Research Ethics Committee of the First Affiliated Hospital of Sun Yat-sen University (2019-004). Five to six-week-old BALB/c-nu/nu mice were obtained commercially (Beijing Vital River Laboratory Animals Co, Beijing, China). Xenografts were generated by injection 3×10^6^ SUNE1/HRE cells subcutaneously in 50 μl PBS into limbs. Tumor size was measured twice per week and volume approximated as (width^2^ × length)/2. Research was initiated according to the protocol indicated when the tumors reached approximately 100 mm^3^ (~6 mm in diameter).

For *in vivo* studies involving low oxygen tension environment, SUNE1/HRE tumor-bearing mice were deposited in a custom-made chamber (VetEquip). A gas mixture containing 10% oxygen and 90% nitrogen was administered for 24 h while age, gender and tumor size matched control mice were maintained in room air. For *in vivo* studies involving irradiation and drugs treatment, 5 Gy of X-ray was given by irradiator (RAD SOURCE RS2000 irradiator, Rad Source Technologies, Inc.) on day 1, 3 and 5 and the drugs (NVP-BEZ235 and DDP) was given on day 1 to 5 at 2 h prior to IR. NVP-BEZ235 (50 mg/kg) was reconstituted in a solution of NMP and PEG300 (1:9) and administered to tumor-bearing mice by oral gavage; whereas DDP was managed via injection intraperitoneally at 5 mg/kg. 80 mg/kg Pimonidazole (Hypoxyprobe) and 25 mg/kg Hoechst 33342 (Sigma-Aldrich) was injected at 120 and 1 min before sacrifice, respectively. Tumors were dissected on 21 days post-IR for immunofluorescent study.

### Fluorescence microscopy

To detect hypoxia-induced GFP expression *in vitro*, SUNE1/HRE cells were treated with 21% O_2_, 0.5% O_2_ or 200μM CoCl_2_ (Sigma-Aldrich) for 24 h, respectively. Hoechst 33342 was used for specifically staining the nuclei. Fluorescent microscopy was used to capture images that showed the intensity of GFP and percentage of cells with GFP.

To measure the number of γH2AX foci, a surrogate of DSB, the cells were irradiated at 5 Gy in presence or absence of NVP-BEZ235 (100 nmol/ml) or DDP (10 μM). The cells were harvested at indicated time post-treatment and processed according to immunofluorescence protocols as described 26. Images were acquired by the LSM 710 laser-scanning confocal microscope (Zeiss) and quantitative image analysis performed by ImageJ.

For detection of endogenous and exogenous hypoxic markers, subcutaneous tumors were cut into 8μm cryosections. Image of HRE-GFP and Hoechst 33342 was first scanned by Zeiss Axio Scan Z1 Slide Scanner (Zeiss). Sections were then rinsed thrice with PBS and fixed by chilled acetone at 4°C for 10 min. Tissues were permeabilized by Triton, blocked with bovine albumin and incubated with primary antibodies at 4°C overnight. Primary antibodies were used as following, FITC-conjugated anti-pimonidazole antibody (Hypoxyprobe) diluted in 1:50; anti-CA9 antibody (Novus) diluted in 1:50; anti-CD31 antibody (Cell Signaling Technology) diluted in 1:50. CA9 and CD31 was examined with an Alexa Fluor 555-conjugated secondary antibody (Life Technologies) for 1.5 h at room temperature in the dark.

### Statistical analysis

DNA repair data were analyzed for significance via ANOVA test by SPSS 19, where p < 0.05 was considered statistically significant. Tumor growth delay curve was depicted by Sigma Plot 12.5. FACS data were analyzed by FlowJo version 10.8.1 software (Tree Star, San Carlos, CA).

## Results

### *In vitro* characterization of human NPC SUNE1/HRE cells

**Figure 1:**
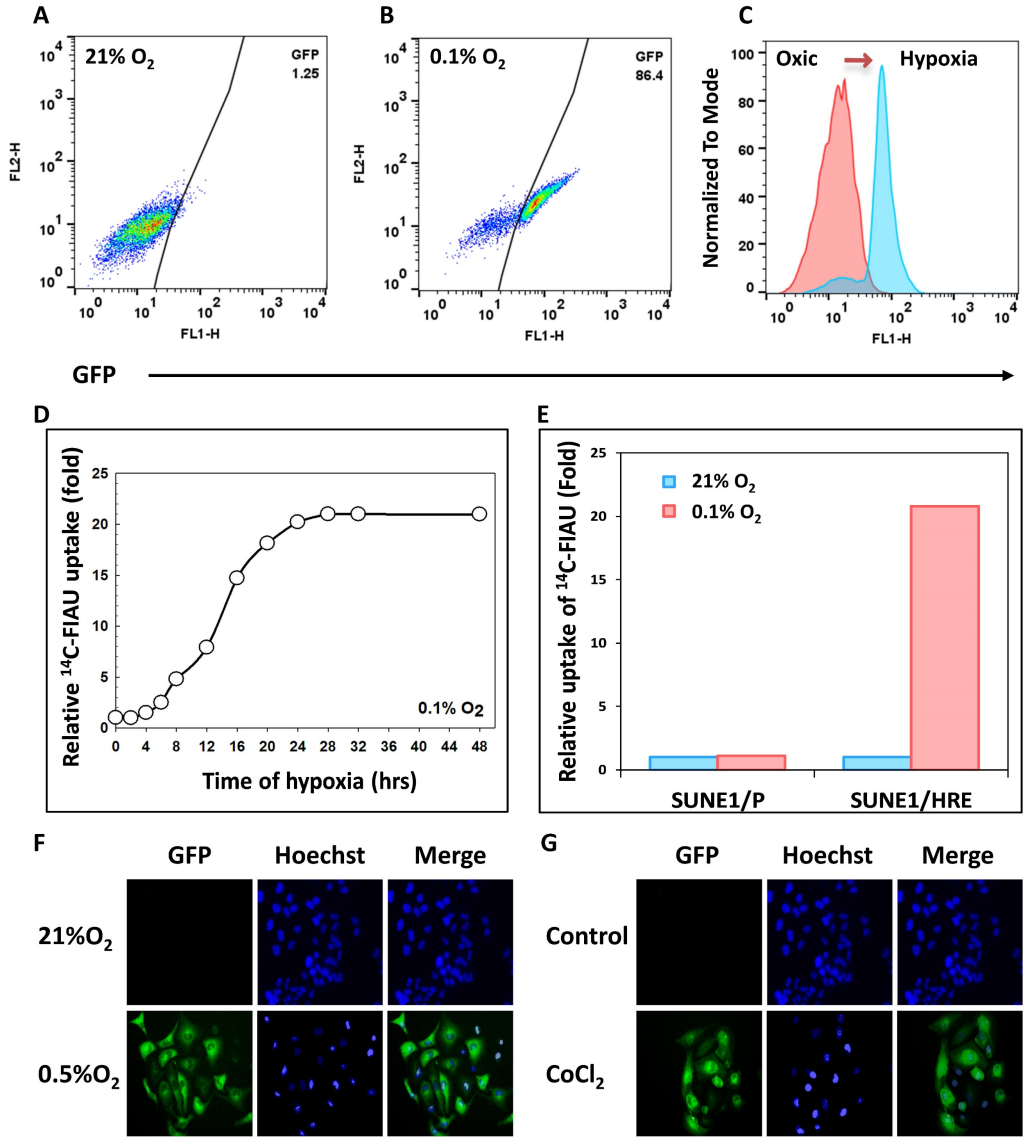
** Validation of 9HRE-TKGFP dual reporter system in transduced NPC SUNE1 cells.** (A) and (B) Detection of hypoxia-induced GFP expression in SUNE1/HRE cells under 21% (A) or 0.1% O_2_ (B) by flow cytometry. (C) Histogram representation of fluorescent intensity of GFP in A and B showing that the expression of GFP was significantly elevated in SUNE1-HRE-TKGFP cells after incubating at 0.1% O_2_ for 24 h. (D) Dynamics of TKGFP expression measured by ^14^C-FIAU accumulation when SUNE1/HRE cells were cultivated at 0.1% O_2_ for the indicated time period. The cells were incubated in the medium containing ^14^C-FIAU (0.025 μCi/mL) for 1h before measurement. (E) TKGFP expression as quantified by ^14^C-FIAU accumulation at least for 1 h after SUNE1/P and SUNE1/HRE cells were exposed to 21% or 0.1% O_2_ for 24 h, respectively. (F-G) Representative fluorescent images of SUNE1/HRE cells incubated in either 0.5% O_2_ or CoCl_2_ (200 μM) for 24 h. Magnification, X300.

As described, the SUNE 1/HRE clonogenic cells were selected by G418 and sorted by FACS for three times to enrich viable cells with hypoxia-induced GFP expression. To confirm the hypoxia-induced GFP expression after enrichment, SUNE1/HRE cells were exposed to 21% O_2_ or 0.1% O_2_ for 24 h. FACS analysis displayed that the proportion of GFP-positive cells significantly increased from 1.25% under normoxia to 86.4% under hypoxia (Fig. 1A and B). In Fig. 1C, histogram plot showed that GFP expression under control of HRE was strikingly upregulated by hypoxia.

Fig. 1D showed that hypoxia-induced expression of TK to phosphorylated FIAU in SUNE1/HRE cells under the treatment of 0.1% O_2_ was time-dependent. The uptake of ^14^C-FIAU was normalized to counting of radiosensitivity/mg protein and further normalized to fold according to the baseline. Upon equilibration with 0.1% O_2_ the accumulation of ^14^C-FIAU was approximately half maximum at 12-16 h and the highest-level exposure of 24 h. The uptake of ^14^C-FIAU was minimal under normoxia in both SUNE1/P and SUNE1/HRE cells. After exposing to 0.1% O_2_, the uptake of ^14^C-FIAU in SUNE1/P cells was similar to the baseline, whereas it was profoundly higher in SUNE1/HRE cells.

We further replicated the FACS analysis using fluorescent microscopy. CoCl_2_ is a common hypoxia-mimetic agent. The cells were cultured at 21% O_2_, 0.5% O_2_ or in the presence of 200 μM CoCl_2_ for 24 h. Green fluorescence was only detected intensively in the hypoxic cells (Fig. 1G and F). HIF1α serves as a surrogate of hypoxia and it was only detected under hypoxic condition, which was consistent with the expression of GFP (Fig. S1).

### *In vivo* characterization of hypoxia-driven gene expression in human NPC SUNE1/HRE tumor model

**Figure 2:**
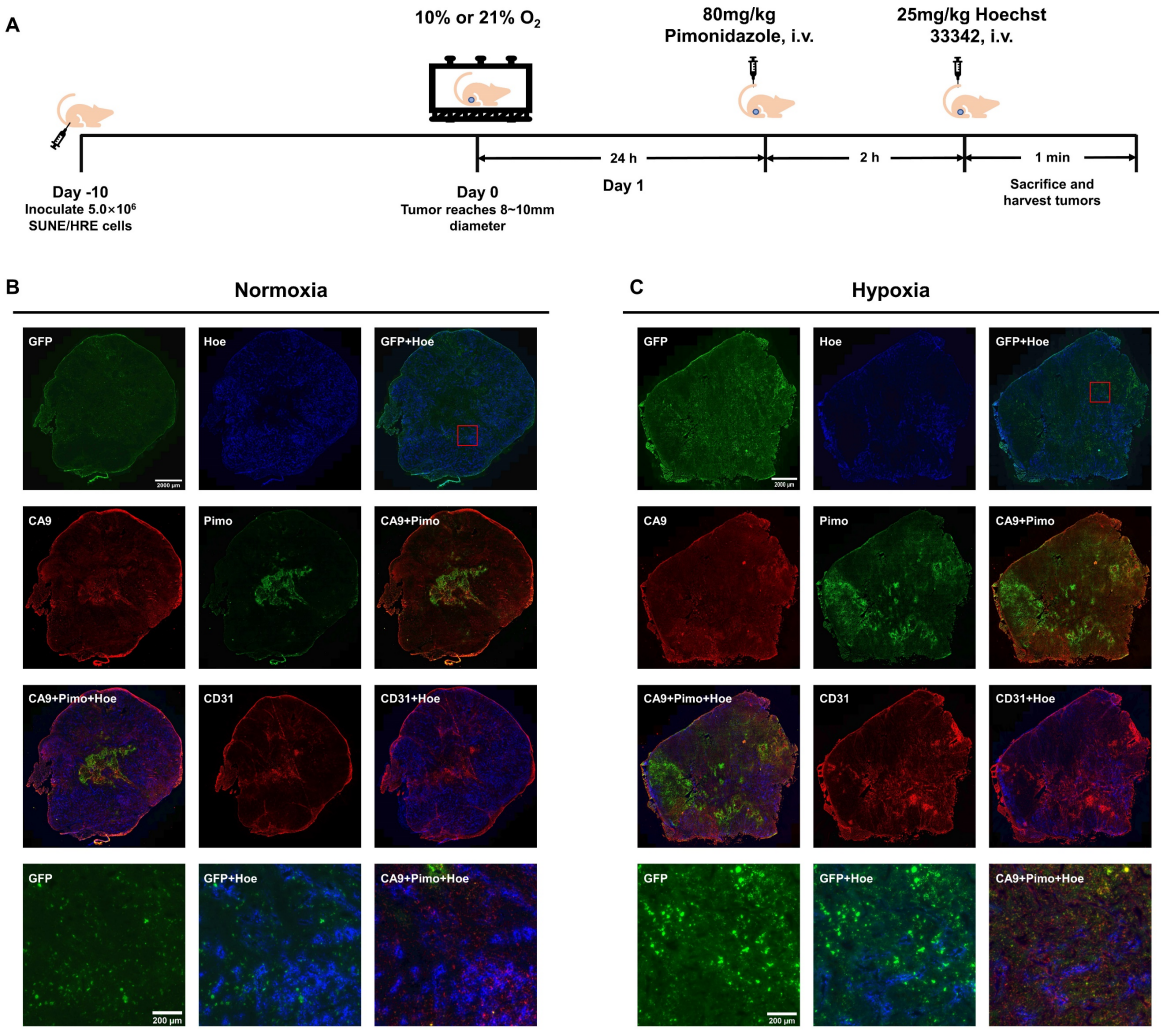
** The evolving spatial biodistribution of hypoxia biomarkers when SUNE1/HRE tumor-bearing mouse breathing 10% O_2_ gas.** (A) Schematic of the experimental design to evaluate hypoxia-induced molecular events. When xenograft was approximately 6 mm in diameter, the animals were randomly assigned into 2 groups, either air breathing (21% O_2_) or hypoxia breathing (10% O_2_) for 24 h. For hypoxia breathing group, the animals were placed in a custom-made chamber with a constant gas supply of 10% O_2_. The animals were then injected with hypoxia marker, pimonidazole (80 mg/kg) via tail vein for 2 h followed by injection of perfusion marker, Hoechst 33342 (25 mg/kg) for 1 min. The animals were sacrificed and tumors were dissected for cryosection. (B) and (C) GFP expression, Hoechst 33342, pimonidazole, CA9 and CD31 images were acquired. Shown are representative image of 3 mice in each treatment group. All images were scanned at X20 magnification. Bar = 2000 µm (global tumor image) and 200 µm (magnified image).

To verify our *in vitro* findings and illustrate the hypoxia-triggered reporter system, we used subcutaneous SUNE1/HRE xenografts and conducted experiment as described in Fig. 2A. The spatial relationship of various biomarkers in xenografts were compared when tumor-bearing mice breathed air or 10% O_2_. These biomarkers included hypoxia reporter gene (GFP), blood perfusion marker (Hoechst 33342), endogenous hypoxic marker (CA9), exogenous hypoxic marker (pimonidazole) and endothelial cells marker (CD31). The level of GFP, pimonidazole and CA9 was low to undetectable in the normoxia group (Fig. 2B); whereas intense GFP, pimonidazole and CA9 fluorescence was visualized in the hypoxia group (Fig. 2C) in comparably smaller tumors.

Co-registered image showed an inverse correlation of biodistribution between areas of GFP and Hoechst 33342 signal, whereas the consistency in biodistributions of the different hypoxic biomarkers was observed. The obvious and unequivocal visualization of the co-registered image showed that pimonidazole and CA9 were trapped in the identical regions within the tumor, predominating regions in reduced density of vessels as indicated by area of low Hoechst 33342 signal, suggesting the contrary relation between hypoxia and blood perfusion. Anti-CD31 staining was used to identify vascularity, functional blood vessels surrounded by well blood perfusion and dysfunctional blood vessels indicated by poor perfusion (acute hypoxia). The three panels in the lower corner are magnified view of GFP, co-registered images of GFP with Hoechst 33342 and CA9 with pimonidazole with Hoechst 33342 providing a detailed view of spatial biodistribution of these markers. Consequently, the HRE-driven reporter system is sufficiently sensitive to locate hypoxia regions within tumors.

### Abrogation of DNA damage repair by irradiation and/or DDP in SUNE1/HRE cells under hypoxia

The radiosensitivity of SUNE1/P and SUNE1/HRE cells was characterized when exposed to IR under normoxia via examining γH2AX foci formation. H2AX is a histone protein, which is quickly phosphorylated to form γH2AX at DSB sites. As a result, γH2AX foci formation is widely used to represent DSB damage due to its sensitivity and specificity 4. The preliminary data were depicted that γH2AX foci formation was observed immediately after 5 Gy of IR exposure, reaching at the maximum at 1 h, then gradually decreased and was similar to that of the cells without exposure to IR (data not shown), indicating that the most majority of DSBs were repaired within 6 h post-IR.

Given that hypoxia acts as one of the major barriers of radiation-induced DNA damage fixation, we examined the IR response of SUNE1/P and SUNE1/HRE cells under hypoxia by detecting DSBs. The cells were maintained in 0.5% O_2_ circumstance for 24 h and then given 5 Gy of X-ray irradiation.

**Figure 3:**
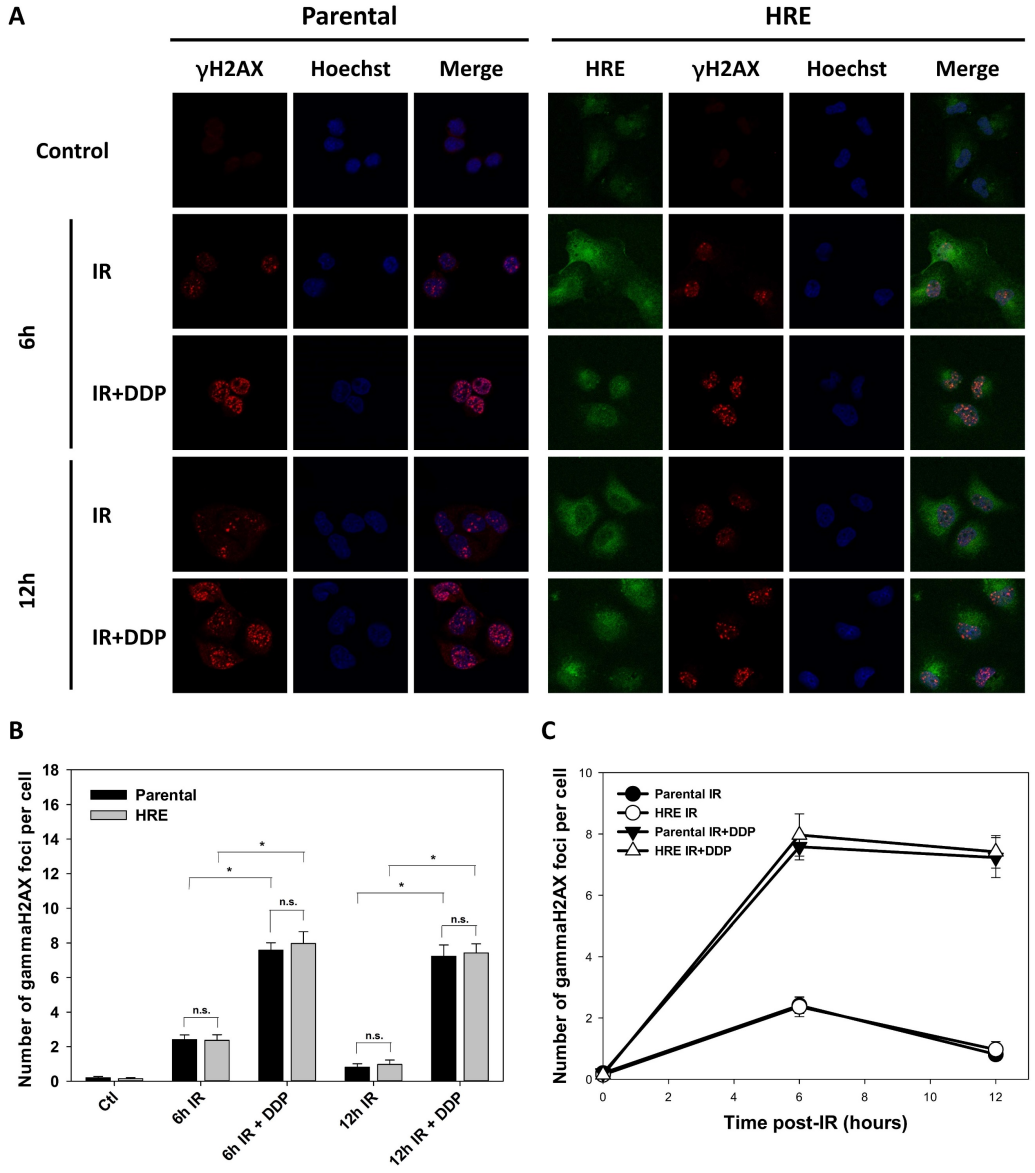
** Comparison of intrinsic radiosensitivity between parental and hypoxic-driven SUNE1 cells.** SUNE1/P and SUNE1/HRE cells were exposed to irradiation of 5 Gy with or without DDP (10 μM) under normoxic or hypoxic condition and harvested at indicated time points after exposure to irradiation for immunofluorescent array of γH2AX foci formation. DSBs were quantified by the number of γH2AX foci. (A) Representative photomicrographs of γH2AX foci (red), Hoechst 33324 (blue), GFP (green) and co-registered image for SUNE1/P and SUNE1/HRE cells (×1000 magnification). (B-C) Comparison of intrinsic radiosensitivity between SUNE1/P and SUNE1/HRE cells by calculating average number of γH2AX foci formation by counting at least 30 nuclei. Data are shown as mean ± SEM. *, p<0.05; n.s., not significant.

As depicted in Fig. 3A, GFP was clearly expressed in the most majority of SUNE1/HRE cells, indicating the cells used for γH2AX foci analysis were severely hypoxic. γH2AX foci were nearly undetectable in control group, whereas radiation-induced γH2AX formation was found at 6- and 12-h post-IR. There was a drastic increase in γH2AX levels in the presence of DDP, a widely used radiosensitizer in multiple types of cancer, especially in NPC, compared to IR alone. DSB repair was substantially delayed, consistent with the radiosensitizing effect of DDP (Fig. 3B and C). The difference of the number of γH2AX foci between two cell lines was not significantly different, suggesting that the build-in hypoxia-driven dual reporter 9xHRE-TKGFP gene did not alter intrinsic radiosensitivity of SUNE1/HRE cells under hypoxia (Fig. 3).

### Combined treatment of DDP and PI3K/mTOR inhibitor suppresses DSB repair

DNA-dependent protein kinase catalytic subunit (DNA-PKcs) represents an essential element during the process of NHEJ repair 27. Ataxia-telangiectasia mutated (ATM)-deficient cells exhibited pronounced HR repair deficiency in G2 phase as a result of RPA and RAD51 foci formation defect 28. The radiosensitizing activity of DDP is correlated to DDP-mediated phosphorylation of ATM which results in prosurvival effect on tumor cells. Therefore, DDP markedly induced radiosensitization of the cells lacking cytoplasmic phosphorylate ATM but not of cells with cytoplasmic p-ATM 29. ATM in upstream of DNA-PK regulates both NHEJ and HR repair pathways. Both of DNA-PKcs and ATM belong to phosphatidylinositol 3-kinase (PI3K) family. Combined treatment of DDP and PI3K pathway inhibitor may exert a synergistic radiosensitizing activity.

**Figure 4:**
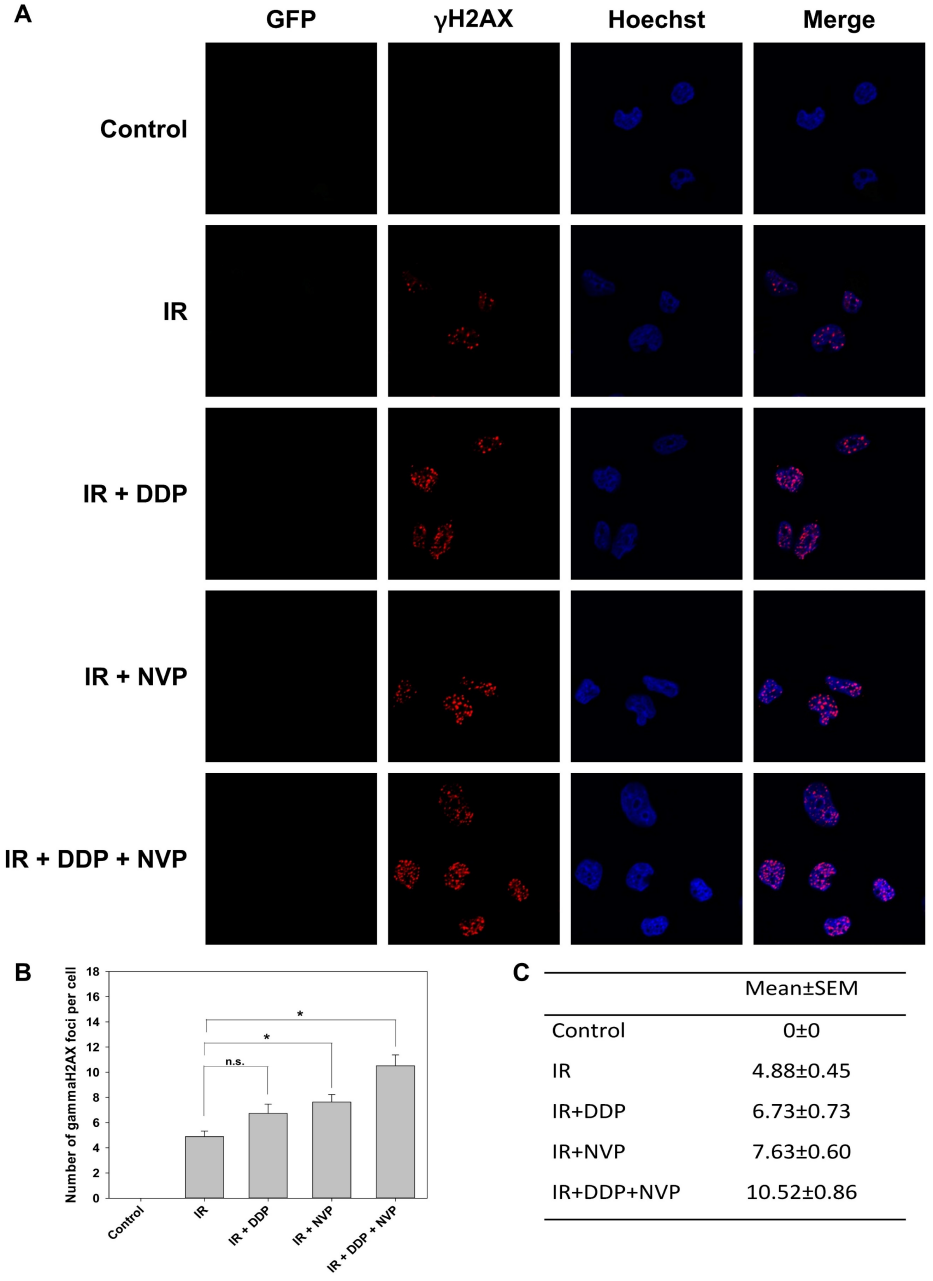
** Synergistic anti-tumoral effect of DDP and PI3K/mTOR dual inhibitor, NVP-BEZ235 to irradiation in SUNE1/HRE cells**. SUNE1/HRE cells were treated with NVP-BEZ235 (100 nmol/ml) and/or DDP (10 µM) 1 h prior to IR of 5 Gy and harvested at 6 h post irradiation for immunofluorescent array of γH2AX foci formation. (A) Representative images of γH2AX foci (red), Hoechst 33342 (blue), GFP (green) and co-registered image for SUNE1/HRE cells (×1000 magnification). (B) Plot and (C) digital number of the average γH2AX foci formation per cell were quantified by automated image quantification from at least 30 nuclei. Data are shown as mean ± SEM. *, p<0.05; n.s., not significant.

To test the hypophysis, a dual PI3K/mTOR inhibitor, NVP-BEZ235, was utilized in our study. It is an imidazo [4,5-c] quinoline derivative whose biochemical characterization was first reported by Maira *et al.* and then compelling evidence has emerged to identify its role in radiosensitization including in head and neck cancer 30-33. Its mechanism was investigated that it radiosensitized glioblastoma cells via compromising activity of ATM and DNA-PKcs 34. SUNE1/HRE cells were irradiated with DDP, NVP-BEZ235 or combined treatments under normoxia. No green fluorescence was observed, which means the cells were oxic (Fig. 4A). Combining NVP-BEZ235 with IR dramatically induced γH2AX foci formation compared to IR alone (Fig. 4B and C). Although no statistically significant difference was observed between IR and IR + DDP group, there was a clear trend that DDP radiosensitized SUNE1/HRE cells. The possible explanation may be the number of analyzed cells were not enough to show a statistic difference. There were markedly more γH2AX foci formation with two compounds compared to single drug, implying an additive effect on DSB repair inhibition (Fig. 4B and C). Our data revealed that the functions of suppressing DSB repair induced by DDP and NVP-BEZ235 were nonredundant.

### The effect of synthetic lethality caused by irradiation in combination with PI3K/mTOR inhibitor and DDP *in vivo*

**Figure 5:**
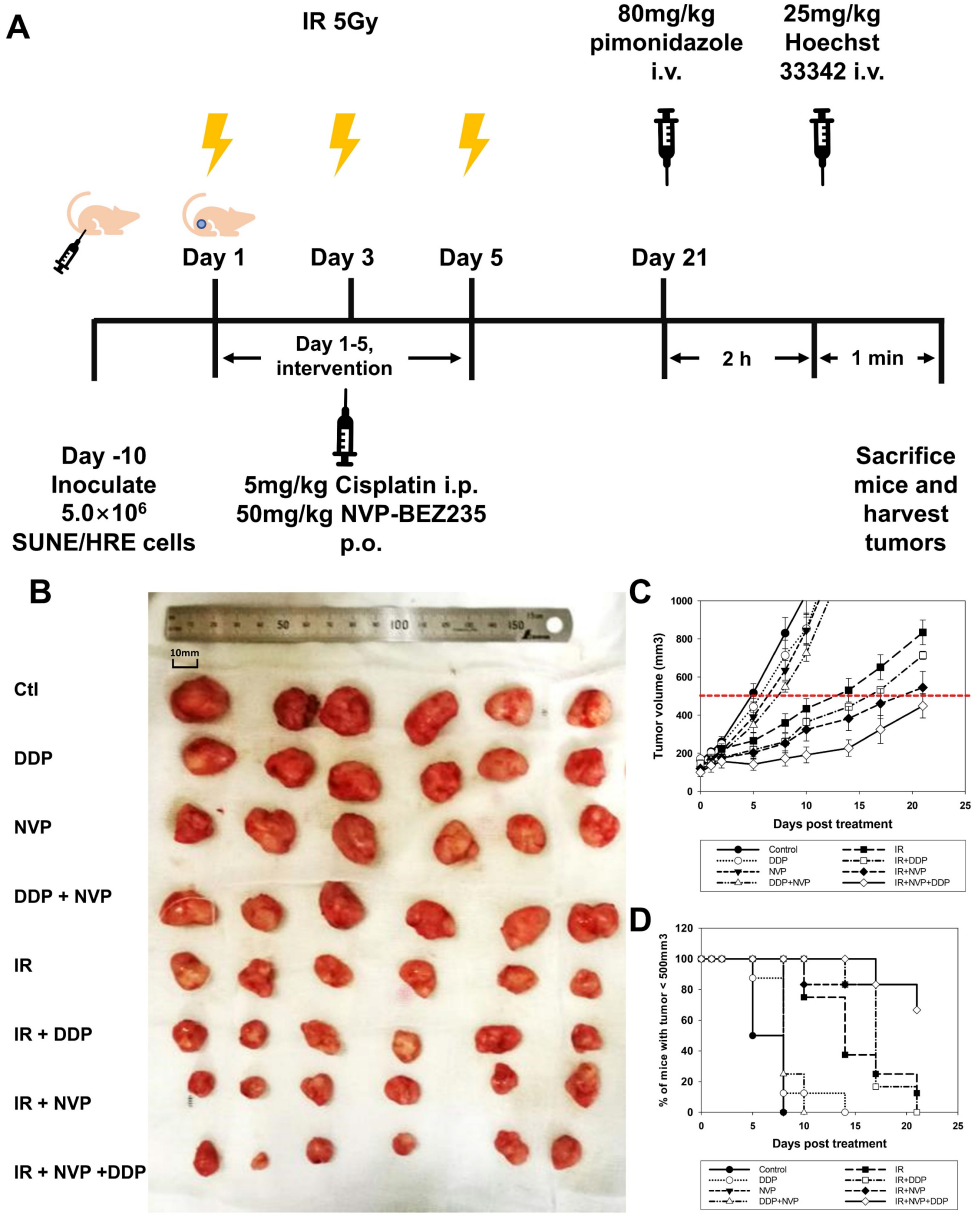
** Tumor growth delay when SUNE1/HRE xenografts-bearing animals irradiated with or without radiosensitizers.** (A) Schematic of the treatment schedule for the IR, DDP, NVP-BEZ235 and their combinations. The xenografts grew to approximately 6 mm in diameter in 10 days, the mice were then randomized into 8 groups, in which they were treated with vehicle, DDP, NVP-BEZ235 and/or in combination with irradiation. The scheme for the drug therapy was as followed: DDP (5 mg/kg), NVP-BEZ235 (50 mg/kg) once daily for 5 days. The schedule for irradiation was three fractions of 5 Gy, which was given every other day. The mice were administered intravenously with the pimonidazole (80 mg/kg) and Hoechst 33342 (25 mg/kg), at 2 h and 1 min before sacrificing, respectively. The xenografts were removed for fresh cryosection and immunofluorescent array. (B) Representative images show xenografts from SUNE1/HRE tumor-bearing mice at 21 days post-treatment. (C) The curve on tumor growth delay by depicting xenograft tumor volumes. Points, mean; bars, SEM; *, p < 0.05. (D) The time for the probability of xenograft reaching the volume of 500 mm^3^.

Our previous studies have shown that blockage of PI3K/mTOR pathways by NVP-BEZ235 promoted the cytotoxic effect via accumulation of unrepaired DSBs (Fig. 4). Therefore, we designed an experiment to validate the hypothesis *in vivo*. The maximal concentration of NVP-BEZ235 in tumor tissue was attained at 1 h after administration and steady-state levels would maintain for 3 to 5 days 30. Considering the pharmacokinetics of NVP-BEZ235, the drug was given orally at 1 h before IR. Both DDP and NVP-BEZ235 were administered once daily for 5 days (Fig. 5A). Moderate dose of DDP or NVP-BEZ235 alone had modest efficacy on shrinking tumor. Tumor volume in DDP + NVP-BEZ235 group was slightly smaller than that in single agent groups. IR displayed moderate efficacy in reduction of tumor volume when compared to the vehicle control. Radiosensitizing activities of DDP and NVP-BEZ235 was observed when combined with irradiation (Fig. 5B and C). The time for the tumor to reach a volume of 500 mm^3^ was evaluated for the tumor growth delay. It took 9, 12, and 16 days in animals treated with IR alone, IR + DDP and IR + NVP-BEZ235, respectively (Fig. 5C and D). Notably, the most potent treatment was triple combination of irradiation, DDP and NVP-BEZ235, which provided an optimal tumor growth delay and reduction of tumor volume, indicating that the therapeutic efficacy of irradiation was significantly enhanced by the combined application of DDP and PI3K/mTOR inhibitor.

### The potential outcomes of antitumoral therapy by imaging tumor microenvironment

**Figure 6:**
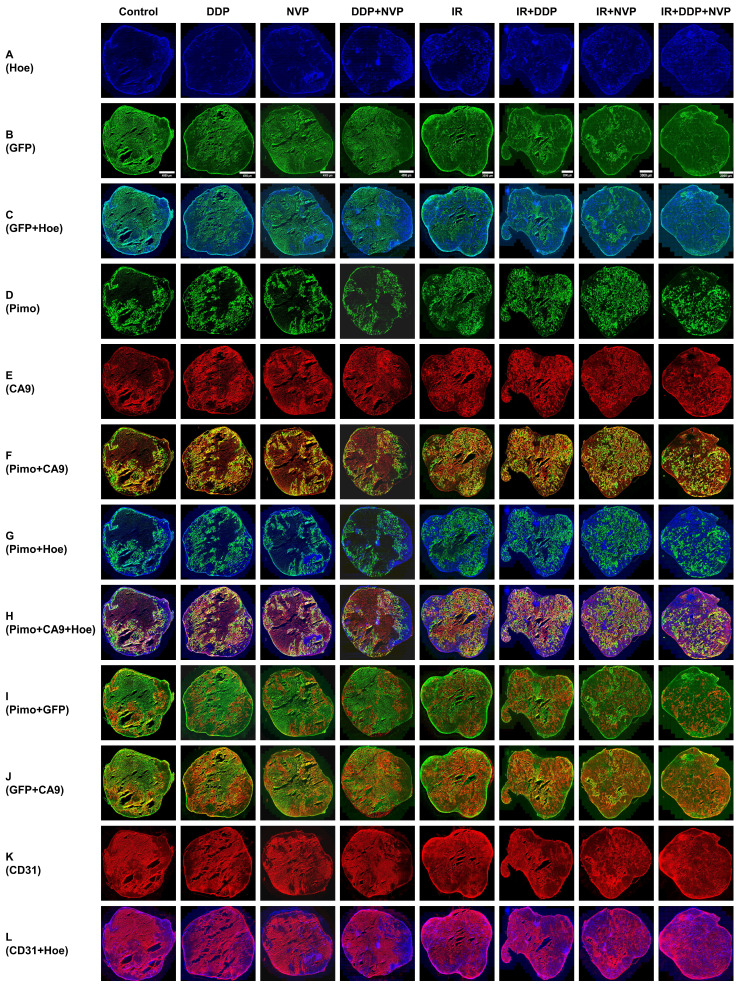
** Multiparametric comparison of hypoxia-related tumor biomarkers in global SUNE1/HRE xenografts.** The SUNE1/HRE xenograft-bearing mice were treated as indicated in figure 5A. The spatial distributions of the multiple hypoxia and blood perfusion markers are shown as follows: (A) Hoechst 33342 (blue); (B) GFP (green); (C) GFP (green) and Hoechst 33342 (blue); (D) pimonidazole (green); (E) CA9 (red); (F) pimonidazole (green), CA9 (red) and the overlay (yellow); (G) pimonidazole (green) and Hoechst (blue); (H) pimonidazole (green), CA9 (red), Hoechst 33342 (blue) and the overlay (yellow); (I) pimonidazole (red), GFP (green) and the overlay (yellow); (J) GFP (green), CA9 (red) and the overlay (yellow); (K) CD31 (red); (L) CD31 (red) and Hoechst 33342 (blue). Shown are representative images of 6 mice in each treatment group. All images were scanned at x 20 magnification. The scale of the bar in the images is as follows, 4000 µm (control, DDP, NVP and DDP+NVP), 3000 µM (IR, IR+DDP and IR+NVP) and 2000 µm (IR+DDP+NVP).

Our preclinical model enables the detection of heterogeneous tumor hypoxic regions in tumor tissues and exploring the treatment efficacy of multiple therapy methods to overcome hypoxia. The area of profound hypoxia was observed in the tumors without IR treatment, whereas tumors were significantly shrunk when exposed to IR, which in turn resulted in restoring normoxia in hypoxic regions. IR combined with DDP or NVP-BEZ235 further reversed tumor hypoxia; IR + DDP + NVP-BEZ235 exhibited the most robust effect on reversal of hypoxia, indicating that the two drugs worked synergistically anti-tumoral efficacy with RT (Fig. 6). Necrotic area with high background and without blood perfusion was observed in the center of xenografts. GFP was expressed in the area of low blood perfusion (Fig. 6A-C). The colocalization of exogenous hypoxic marker pimonidazole and endogenous marker CA9 was concordant (Fig. 6D-F). Fig. S2 provides a more detailed comparison and spatial distribution of the different biomarkers in magnified views of a region from a SUNE1/HRE xenografts after various treatments.

The accumulation of pimonidazole was modest in well-perfused area within tumor, and high in poor-perfused area; as the accumulation of CA9 (Fig. 6G and H). Fig. 6I and J illustrates the patterns of GFP with CA9 and pimonidazole, respectively. The GFP and pimonidazole were almost identical, but there was some discrepancy between them at micro-regional level probably due to varying concentration of pO_2_ (Fig. 6I). The GFP was also overlapped with CA9 (Fig. 6J). We subsequently determined the vascularity by visualizing density and colocalization of Hoechst 33342 and CD31 staining. We found more functional blood vessels (co-stained with Hoechst 33342 and anti-CD31) in the irradiated tumors. Of interest, the non-irradiated tumors but treated with NVP-BEZ235 regardless of tumor size contained more functional blood vessels compared to control or the one treated with DDP alone. Meanwhile, there were plenty of obstructed blood vessels (Hoechst 33342 negative and CD31 positive staining) in the tumors without treatment or treated with DDP alone (Fig. 6K-L). Therefore, our observations not only underline the importance of HRE-driven reporter system for imaging tumor hypoxia, but also indicate a potential tool for investigating effective therapeutic modality for NPC.

## Discussion

NPC is an endemic cancer in southern China with high propensities to metastasize to distant sites affecting up to one-third patients in high-risk subgroups 35, 36. With advanced modern RT technique and comprehensive multidisciplinary treatment, there has been reported fewer locoregional recurrences for non-metastatic NPC. However, approximately 10% of NPC relapses at the primary and/or regional site 22. Hypoxia, a general feature of solid tumors, may contribute to the locoregional failure of NPC and has critical implications for the administration of cancer patients. Thus, the present study aims to develop an HRE-driven reporter gene model depending on the hypoxia-driven molecular “switch” i.e., the expression of HIF-1 to evaluate and quantify hypoxia in NPC. A ~50 bp sequence of human EPO gene functions as HRE, which induces transactivation function of HIF1α 37. The level of the fusion gene (9xHRE-TKGFP) serves as a reference of hypoxia-facilitated HIF-1α stabilization/HIF-1 transactivation. The SUNE1/HRE tumor model enables us to visualize the correlation between tumor hypoxia and treatment outcome. We examined the sensitivity and specificity of SUNE1/HRE in response to hypoxia. ^14^C-FIAU and GFP was detectable after 4 h exposed to 0.1% and 0.5% O_2_, respectively (Fig. 1D and F). ^14^C-FIAU was only accumulated in hypoxic cells but not in normoxic cells, while GFP was only expressed under hypoxia (Fig. 1E and F). Consistent with our previous data, CoCl_2_ had similar effect on inducing GFP expression to hypoxia (Fig. 1G) 38. Our hypoxia research model in NPC contains hypoxia-driven dual reporter 9xHRE-HSV1-TKGFP fusion gene, imaging system of which can accurately and reliably map hypoxia distribution in NPC. Build-in hypoxia-driven dual reporter does not influence intrinsic radiation sensitivity of SUNE1 parental cells (Fig. 3).

It is a useful tool to study the hypoxia-mediated radioresistance with its excellent spatial resolution in optical imaging. Inhibition of DNA repair could be an underlying strategy to overcome the resistance to chemo-RT. Based on the result that TKGFP expression is induced by hypoxia, it is inferred that the locoregional accumulation of GFP in NPC xenografts corresponds to hypoxic volumes in particular when oxygenation level for tumor-bearing animal is manipulated to 10%. By visual inspection, consistent patterns of hypoxic regions were observed among GFP, CA9 and pimonidazole, respectively (Fig. 2 and 6). Despite correlation between CA9 and pimonidazole is intermediate, CA9 fraction is markedly higher in pimonidazole positive area implying severely hypoxic sites 39. In our results, GFP was principally expressed in pimonidazole and CA9 double positive area (Fig. 6). One presumable explanation for the phenomenon is that TKGFP reporter gene is triggered by severe hypoxia. However, all the hypoxic indicators showed opposite relation with blood perfusion marker, Hoechst 33342.

Overcoming hypoxia and suppressing DNA repair are two main therapeutic approaches to improve RT effectiveness. Hypoxia image guided radiotherapy (HIGRT) via non-invasive imaging method has been carried out to escalate the dose to radioresistant hypoxic foci to increase the tumor control probability 40-42. Our previous studies revealed that the spatial distribution of the two PET tracer, ^124^I-FIAU and ^18^F-FMISO, were identical in prostate and colorectal cancer hypoxia models 16, 17. Our study has for the first time developed the NPC SUNE1/HRE reported system that would detect tumor hypoxia by both non-invasive (uptake of ^14^C-FIAU) and optical (expression of GFP) methods. It provides a powerful tool to address the mechanism of tumor hypoxia on therapeutic intervention in NPC. Our results (Fig. 5 and 6) are in agreement with recent experimental studies that hypoxia-induced radioresistance can be reversed by hypoxic modifiers 19, 43.

DDP-based concomitant chemo-RT is the standard of modality for locoregionally advanced NPC with excellent locoregional tumor control rate 44. There would be a synergistic efficacy between DDP and PI3K pathway inhibitor. Dual therapy of DNA-PK inhibitor and carbon ion irradiation exhibited robust capacity of eradicating radioresistant hypoxic tumor cells 45. NVP-BEZ235, a novel PI3K/mTOR inhibitor, impedes HIF1α synthesis which in turn improves the cytotoxic effect under hypoxia 46. Our researches have been demonstrated that NVP-BEZ235 exhibited a strongly supra-additive effect of irradiation especially when combined with DDP by suppressing DSB repair (Fig. 4 and 5); whereas NVP-BEZ235 improved blood perfusion regardless of irradiation (Fig. 6) by normalizing tumor vasculature to alleviate hypoxia and increase oxygenation 47. The mechanism of radiosensitizing effect of NVP-BEZ235 is comprehensive and merited for further study.

Our preliminary data have been shown that hypoxia could induced expression of VISTA and Arginase 1, a key immune regulator; the knockout of immunosuppressive checkpoint VISTA in MDSCs would lead to changes in HIF1α pathway-related gene signature (data unpublished). Our model would be a powerful tool to map tumor immune microenvironment (TIME) and hypoxia region for further study. Solid tumors may exhibit in a hypoxic TIME that conceals a series of biological factors through diverse environmental stress, which manipulates immunological characteristics within tumor via 1) inhibiting T cell proliferation and cellular cytotoxicity, and promoting T cells apoptosis, 2) suppressing activation and cytotoxic effects of NK cells, 3) impairing differentiation, maturation and migration of dendritic cells, 4) driving immunosuppressive cells including regulatory T cell (Treg) and myeloid-derived suppressor cells (MDSCs) into TME, 5) upregulating expression of inhibitory immune checkpoints on both tumor cells and immune cells 12, 48, 49.

Hypoxic TME is implicated in promoting tumor invasiveness by supporting cancer cells and immunosuppressive cells with a metabolic benefit 50. This is relevant for orchestrating tumor eradication following RT, as potential systemic tumor control relies on an effective immune system. Effects of RT on TIME are complex and dual: 1) releasing chemokines to attract inflammatory cells to TME, liberating tumor antigens to prime T cells and promote cytotoxicity of T and NK cells; 2) facilitating the accumulation of immunosuppressive cells in TME and M2 polarization of tumor-associated macrophages 51, 52. A phase 2 clinical trial showed that stereotactic ablative radiotherapy (SABR) with immunotherapy dramatically improved event-free survival in patients with early-stage NSCLC comparted to SABR alone. Monitoring hypoxic condition may enable the prediction of the treatment outcome of RT and immunotherapy. An MRI-based technology using contrast-amplifying nanoprobes senses tumor acidosis for precisely quantifying hypoxia in the tumor, which is inversely related to therapeutic effect of RT and immunotherapy 53.

However, there are few studies on immunoradiotherapy focusing on hypoxic tumors; the rationale of alleviating hypoxia in combined therapy to improve tumor control needs to be further investigated. Humanized mice enable recapitulation human immune system and resultant tumor microenvironment, in which implanting our NPC xenograft model with functional hypoxia imaging provides a clinically relevant method to non-invasively show hypoxia in tumors and dynamically monitor spatial relationship between hypoxic cancer cells and tumor-infiltrating cells during the treatment. Combining all the strategies would help us further understand the mechanism of evasion of immunosurveillance of hypoxic cancer cells and determine the optimal sequence during the combination of regimens, i.e., RT, chemotherapy, hypoxia modifier and immuno-modulatory approaches.

## Conclusions

In summary, we have developed and characterized a novel human NPC model containing a hypoxia-inducible dual reporter HSV1-TKGFP system to investigate tumor hypoxia *in vitro* and *in vivo*. Tumor hypoxia was measured and confirmed by exogenous and endogenous hypoxic surrogates, yielding excellent similarity. Using this system makes possible the identification of the mechanism of DDP and/or PI3K/mTOR inhibitor-mediated radiosensitization. The hypoxia-driven human NPC model would be further applied in the research of tumor microenvironment with several other similar models of radio-sensitive and -resistant NPC cell lines on synergetic efficacy between radiation and other treatment modalities, i.e., chemotherapy, hypoxia modifier, immunotherapy, and targeted therapy.

## Supplementary Material

Supplementary figures.

## Abbreviations

NPC: nasopharyngeal carcinoma
DSB: DNA double strand breakage
DDP: cis-diamminedichloroplatinum, cisplatin
HRE: hypoxia-responsive element
HSV1-TK: human herpes simplex virus 1-thymidine kinase
GFP: green fluorescent protein
HSV1-TKGFP: fusion gene of human herpes simplex virus 1-thymidine kinase and enhanced green fluorescent protein
^14^C-FIAU: carbon-14 labeled-2'-Deoxy-2'-fluoro-h-D-arabinofuranosyl-5-iodouracil
FACS: flowcytometry activated cell sorting
NaOH: sodium hydroxide
HCl: hydrochloric acid
BSA array: bovine serum albumin array
SUNE1/P cells: parental NPC SUNE1 cells
SUNE1/HRE cells: hypoxia-driven human NPC cells which contain HRE promoter and HSV1-TKGFP fusion gene
Gy: gray
CoCl_2_: copper chloride
PBS: phosphate-buffered saline
FITC: fluorescein isothiocyanate
IR: irradiation
NHEJ: Non-homologous end joining
HR: homologous recombination
RT: radiotherapy
TME: tumor microenvironment
HIF: hypoxia inducible factor
CA9: carbonic anhydrase 9
IR: ionizing radiation
chemo-RT: chemoradiotherapy
FBS: fetal bovine serum
cRPMI: complete RPMI
DNA-PKcs: DNA-dependent protein kinase catalytic subunit
PI3K/mTOR: phosphoinositol 3-kinase/mammalian target of rapamycin
ATM: Ataxia-telangiectasia mutated
PET/CT: positron emission tomography/computer tomography
HIGRT: hypoxia image guided radiotherapy
Treg: regulatory T cell
MDSC: myeloid-derived suppressor cells
TIME: tumor immune microenvironment
SABR: stereotactic ablative radiotherapy
NSCLC: non-small-cell lung cancer
