# Supplementary material for: Establishment and Application of Novel Hypoxia-driven Dual-reporter Model to Investigate Hypoxic Impact on Radiation Sensitivity in Human Nasopharyngeal Carcinoma Xenografts
Source: J Cancer. 2024 Jun 11;15(13):4345–59. doi: 10.7150/jca.96378 (PMC11212076; doi:10.7150/jca.96378)
Supplement: Supplementary file 1 — Supplementary figures. [file jcav15p4345s1.pdf]

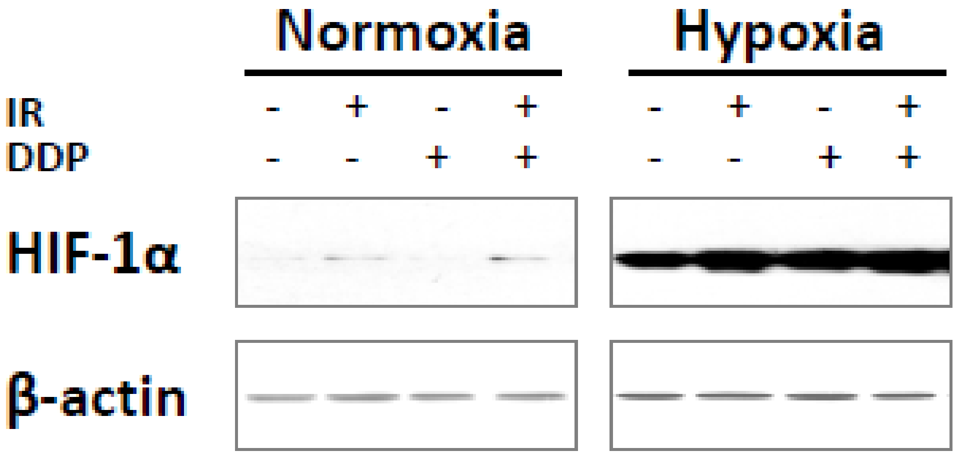

Figure S1. The expression of HIF1α expression in SUNE1/HRE cells under normoxia and hypoxia. $CoCl_2$ was used to mimic hypoxic condition *in vitro* and SUNE-1/HRE cells were treated with or without 200μM $CoCl_2$, and then exposed to IR of 5 Gy in presence or absence of 20μM cisplatin. Cells were harvest at 24 hours post-treatment and the protein level was detected and quantified by Western blot. β-actin was used as the loading control. Representative image from three independent experiments is shown.

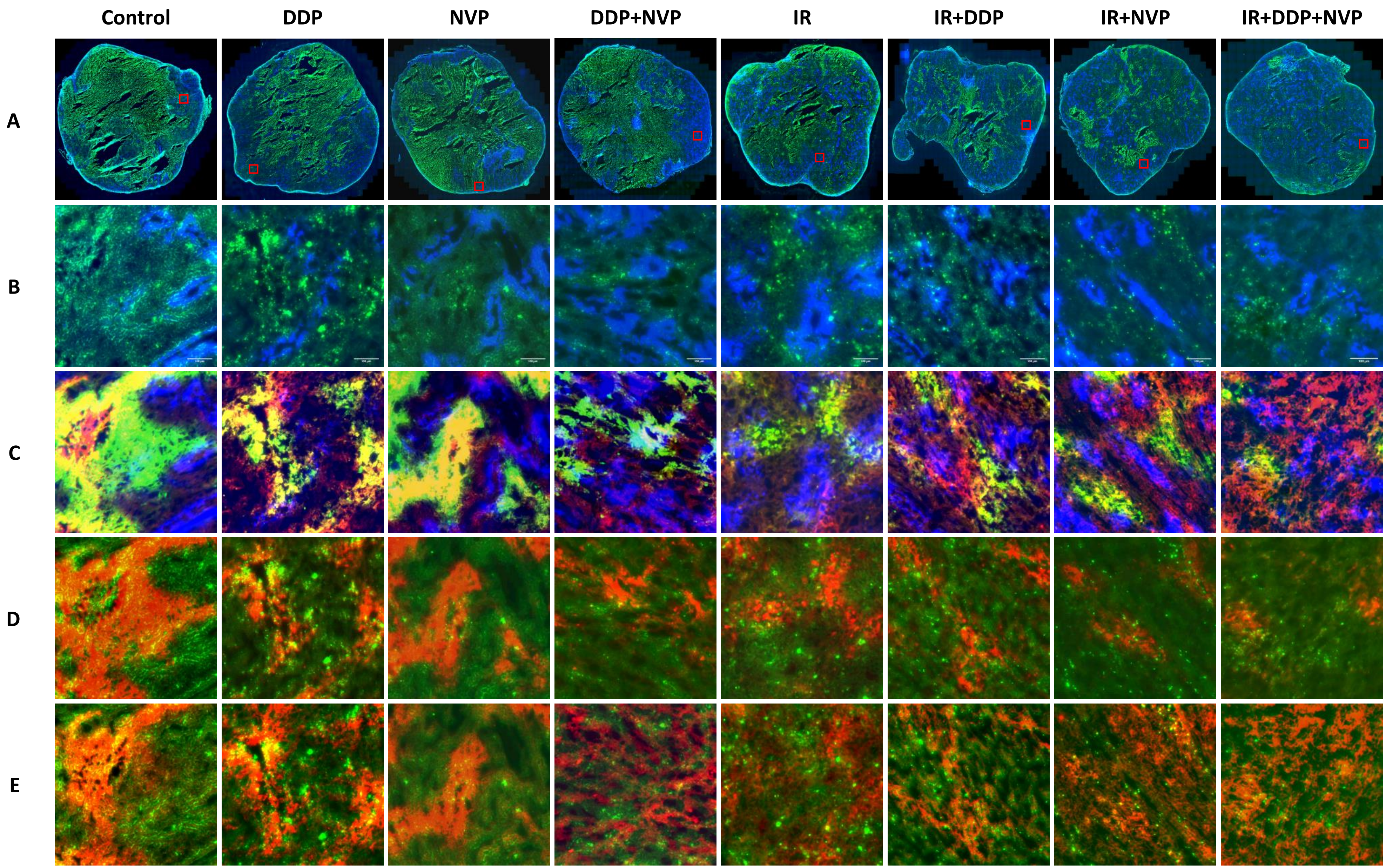

Figure S2. Detailed comparison of various biomarkers in SUNE1/HRE xenografts. Representative images were obtained from the magnified parts in Figure 6. GFP-expressing cells are preferentially observed at a distance to blood vessels. (A) Regions of interest marked by red boxes in GFP + Hoe in Figure 6; (B) GFP (green) and Hoechst (blue); (C) Pimonidazole (green), CA9 (red), Hoechst (blue) and the overlay (yellow); (D) Pimonidazole (red), GFP (green) and the overlay (yellow); (E) GFP (green), CA9 (red) and the overlay (yellow); Bar = 100µm.
